# Supplementary material for: Venomix: a simple bioinformatic pipeline for identifying and characterizing toxin gene candidates from transcriptomic data
Source: PeerJ. 2018 Jul 31;6:e5361. doi: 10.7717/peerj.5361 (PMC6074769; doi:10.7717/peerj.5361)
Supplement: Supplemental Information 3 [file peerj-06-5361-s003.gz › FinalOutput_E-6/Peroxiredoxin-4_1/finaltree.pdf]

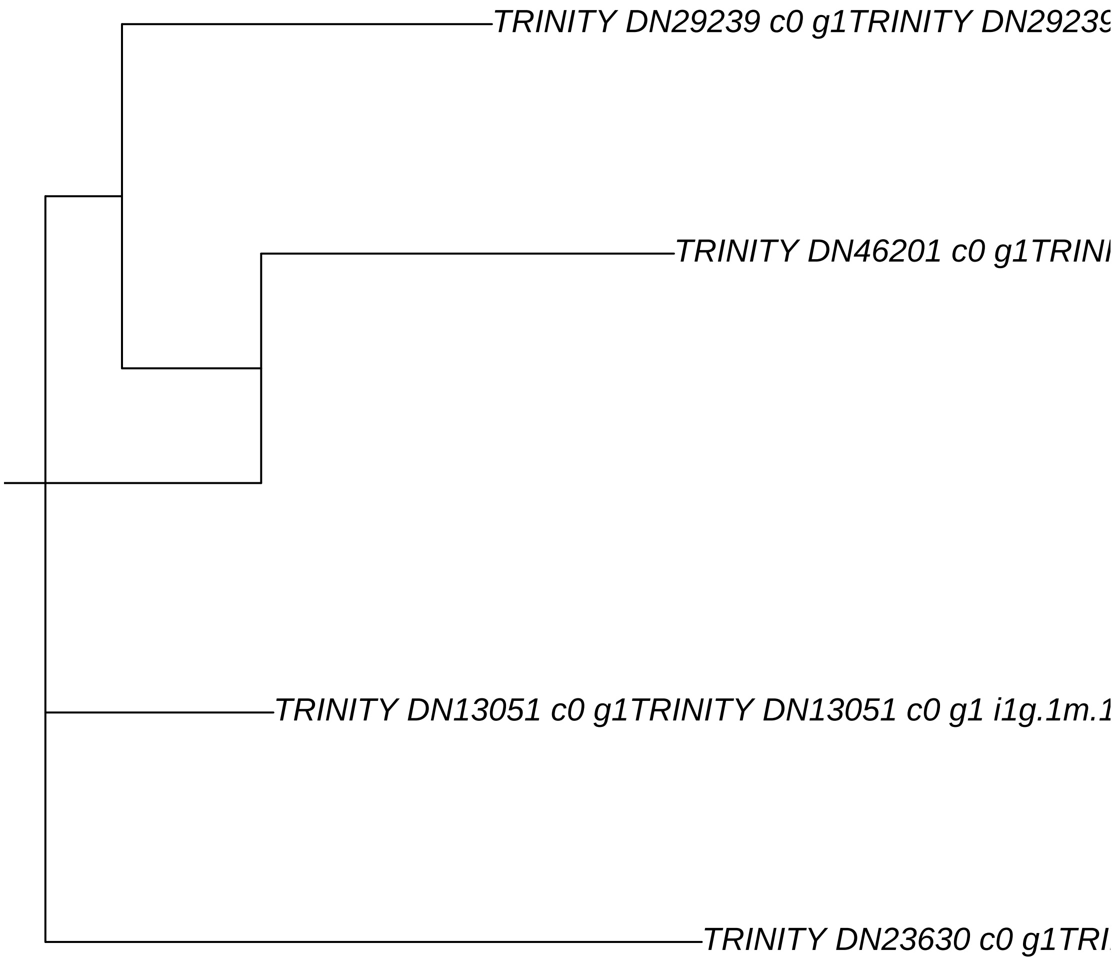
TRINITY DN29239 c0 g1TRINITY DN29239
TRINITY DN46201 c0 g1TRINI
TRINITY DN13051 c0 g1TRINITY DN13051 c0 g1 i1g.1m.1
TRINITY DN23630 c0 g1TRI
